# Heterogeneity of miRNAs in Salivary Gland Neoplasms: An Exploratory Pilot Study

**DOI:** 10.1111/jop.70150

**Published:** 2026-05-25

**Authors:** Dandara Andrade de Santana, Poliana Ramos Braga, Daniel Araki Ribeiro, Patricia Ramos Cury, Ana Carolina Velasco Pondé de Sena, Flávia Caló de Aquino Xavier, Fernando Augusto Soares, Iguaracyra Barreto de Araújo, Fabio Albuquerque Marchi, Valéria Souza Freitas, Cláudia Malheiros Coutinho‐Camillo, Jean Nunes dos Santos

**Affiliations:** ^1^ Post‐Doctoral Fellow, School of Dentistry Federal University of Bahia Salvador Bahia Brazil; ^2^ Postgraduate Program in Dentistry and Health, School of Dentistry Federal University of Bahia Salvador Bahia Brazil; ^3^ Department of Biosciences, Institute of Health and Society Federal University of São Paulo São Paulo Brazil; ^4^ Laboratory of Oral and Maxillofacial Pathology, School of Dentistry Federal University of Bahia Salvador Bahia Brazil; ^5^ Pathology Division Rede D'or Hospital Network São Paulo Brazil; ^6^ Department of Pathology and Legal Medicine, Medical School Federal University of Bahia Salvador Bahia Brazil; ^7^ Department of Head and Neck Surgery University of São Paulo Medical School São Paulo Brazil; ^8^ Center for Translational Research in Oncology, Cancer Institute of the State of São Paulo (ICESP) São Paulo Brazil; ^9^ Department of Health Feira de Santana State University Feira de Santana Bahia Brazil; ^10^ International Research Center A.C. Camargo Cancer Center São Paulo São Paulo Brazil

**Keywords:** cancer, microRNA, salivary gland neoplasms

## Abstract

**Introduction:**

MicroRNAs (miRNAs) are small RNAs that have been associated with tumourigenesis and tumour progression and that play an important role in the pathogenesis of salivary gland neoplasms. The aim of this study was to evaluate the global miRNA expression profile in a sample of salivary gland neoplasms.

**Methods:**

Cases of pleomorphic adenoma (*n* = 4), adenoid cystic carcinoma (*n* = 4) and mucoepidermoid carcinoma (*n* = 4) were studied by real‐time RT‐PCR.

**Results:**

The main up‐regulated miRNAs were hsa‐miR‐373, hsa‐miR‐675, hsa‐miR‐508, hsa‐miR‐1290 and hsa‐miR−483‐3p, while hsa‐miR‐105 and hsa‐let‐7f‐1 were among the most down‐regulated miRNAs compared to the normal salivary gland. Hsa‐miR‐106b, hsa‐miR‐142‐5p, hsa‐miR‐622, hsa‐miR‐642, hsa‐miR‐574‐3p and hsa‐miR‐486 were significantly differentially expressed (*p* < 0.05) when each neoplasm and normal glandular tissue were compared.

**Conclusion:**

This study described the global molecular signature of miRNAs in benign and malignant salivary gland neoplasms and provided preliminary evidence of miRNA dysregulation, suggesting a potential association between specific miRNA signatures and molecular pathways implicated in tumour progression.

## 1 Introduction

MicroRNAs (miRNAs) are small non‐coding RNAs that play a crucial role in the post‐transcriptional regulation of gene expression, mediating processes such as messenger RNA degradation and translation inhibition [1]. The aberrant expression of miRNAs is related to processes such as tumourigenesis and cancer initiation and progression, with these RNAs participating in cancer biology either as oncogenes or as tumour suppressor agents [2]. These regulatory RNAs have been identified in a range of cancers, in which they are particularly important for the modulation of essential cellular processes such as transcription, proliferation, differentiation and apoptosis. In salivary gland neoplasms, miRNAs play an important role in the pathogenesis of the tumour, modulating the cellular biology and biological behaviour of each entity [3].

Salivary gland neoplasms are rare head and neck tumours that are characterised by distinct clinical behaviour and wide biological diversity, including genetic aspects. Despite the phenotypic heterogeneity, the identification of common genetic alterations in different types of salivary gland neoplasms has greatly expanded our understanding of their pathogenesis, contributing to the diagnosis and treatment of these tumours [4]. Within this context, the study of miRNAs [5, 6, 7] may represent a promising strategy to unravel these molecular mechanisms, providing insights into the diagnosis of salivary gland tumours.

In a previous study, we demonstrated that miR‐17, miR‐132, miR‐195 and miR‐221 appear to play an important role as tumour suppressors in salivary gland tumours [5]. However, to our knowledge, few studies have addressed the global expression of miRNAs [6]. Nevertheless, Denaro et al. (2019), who investigated 798 microRNAs in salivary gland tumours, identified 46 miRNAs that were differentially expressed between malignant and benign tumours [7]. These findings suggest the possible development of target‐specific therapies involving the different pathways of tumourigenesis in this group of tumours. Other authors evaluated the expression of 798 miRNAs in 19 adenoid cystic carcinoma (ACC) samples and associated the findings with the aggressive behaviour of the tumour. The results showed overexpression of miR‐29c, miR‐140, miR‐195, miR‐24, miR‐143 and miR‐21 in samples with perineural invasion [6].

Despite increasing knowledge of the role of miRNAs in cancer biology, studies investigating the global expression of these biomarkers in salivary gland neoplasms are still scarce. Therefore, this study investigated the global expression profile of miRNAs in pleomorphic adenoma (PA), mucoepidermoid carcinoma (MEC) and ACC in order to contribute to a better understanding of their regulatory role in the development of salivary gland neoplasms, since these miRNAs could be used as therapeutic targets and diagnostic markers.

## 2 Materials and Methods

### 2.1 Sample Selection

The protocol of the study was approved by the Ethics Committee of the School of Dentistry, Federal University of Bahia (FOUFBA; Opinion number 487.2995.1). The sample was composed of 12 cases of salivary gland neoplasms, including four PAs, four ACCs and four MECs. The control group consisted of two samples of histologically normal salivary gland tissue obtained from non‐neoplastic surgical specimens and processed under identical experimental conditions. The sample of this study was composed of specimens obtained from FOUFBA.

Formalin‐fixed, paraffin‐embedded (FFPE) surgical specimens with preserved tissues were selected. Cases without biologically preserved tissue, paraffin blocks containing little material, and cases whose clinical data were unavailable and/or incomplete were excluded from the study. The medical records of the patients and biopsy forms were used as original sources for the collection of clinical‐pathological data, as shown in Table S1. Written informed consent was obtained from each subject following the Declaration of Helsinki.

For diagnostic review and selection of representative areas of each tumour specimen, 5‐μm‐thick sections were stained with haematoxylin‐eosin and analysed by an experienced oral pathologist, who described the most representative features of the tumours and performed histological grading based on the World Health Organization classification [8].

### 2.2 Total RNA Extraction and cDNA Synthesis

Total RNA was extracted from four 5‐μm‐thick sections of paraffin‐embedded tissue using the miRNeasy FFPE Kit (Qiagen, Tokyo, Japan) according to the manufacturer's instructions. To measure the quantity and quality of the extracted RNA, 1.5 μL of total RNA extracted from each sample was analysed in a spectrophotometer (NanoDrop, Life Technologies Thermo Scientific, Wilmington, USA). Complementary DNA (cDNA) was synthesised from 3 μL (300 ng) of extracted RNA by reverse transcription using the TaqMan MicroRNA RT Reverse Transcription Kit (Life Technologies, Thermo Fisher Scientific, Wilmington, USA) and Megaplex RT Primers, Human Pool Set (Thermo Fisher Scientific) according to the manufacturer's protocols.

### 2.3 cDNA Amplification

To uniformly increase specific cDNA targets and to improve the capacity of detecting miRNA expression in the samples, cDNA pre‐amplification was performed using the TaqMan PreAmp Master Mix and Megaplex PreAmp Primers, Human Pool Set v3.0 (both from Life Technologies, Thermo Fisher Scientific). Next, quantitative real‐time PCR amplification of predefined human miRNA targets was performed on a 7900HT Fast Real‐Time PCR System (Applied Biosystems—Life Technologies, USA) using the TaqMan Universal PCR Master Mix (Life Technologies, Thermo Fisher Scientific) according to the manufacturer's recommendations.

### 2.4 MicroRNA Array

The miRNA expression profile was determined individually for each sample using the predefined human miRNA targets of the TaqMan MicroRNA Array v3.0 platform (Applied Biosystems—Life Technologies, USA), which consists of two independent microfluidic cards, the Human MicroRNA A Card v3.0 and the Human MicroRNA B Card v3.0 (Applied Biosystems—Life Technologies, USA). Each card contains specific oligonucleotides and TaqMan probes sufficient for the analysis of 377 human miRNA targets, in addition to four control genes, for a total of 754 predefined human miRNAs on both cards. cDNA was quantified in a 7900HT Fast Real‐Time PCR System (Applied Biosystems—Life Technologies, USA). The cycle at which the amplification curve exceeded the fluorescence detection threshold (threshold cycle, CT) was determined and used for gene expression quantification. The hsa‐miR‐28 gene was selected as endogenous control using the NormFinder equation (MOMA—Department of Molecular Medicine, Denmark).

### 2.5 Analysis of microRNA Data

For each miRNA studied, quantification was performed based on ΔCT, which represents the difference between the expression of the target miRNA and that of the endogenous control in a given sample. Relative expression levels were calculated using the 2^−ΔΔ*Ct*^ method, in which ΔΔCT is the difference between the ΔCT values of the tumour and control samples. Amplification curves were analysed using the RQ Manager 1.2 software (Applied Biosystems—Life Technologies, USA) to assess reaction quality and calculate CT values. MicroRNAs were considered differentially expressed when they met the following criteria: *p* value < 0.05, false discovery rate (FDR) < 0.1, and absolute Log fold change (LogFC) greater than 2.

The Student *t*‐test, chi‐squared test and analysis of variance (ANOVA) were applied to compare expression profiles across the groups of interest, with statistical significance defined as *p* value < 0.05 and FDR < 0.1.

## 3 Results

### 3.1 Differential miRNA Expression Between Salivary Gland Neoplasms and Normal Salivary Gland Tissue

Comparison of the three salivary gland neoplasms (PA [*n* = 4], ACC [*n* = 4] and MEC [*n* = 4]) with normal salivary gland tissues (*n* = 2) revealed significant differential expression (*p* < 0.05) of 29 miRNAs, as shown in Table S2. Of these, 27 (93.1%) miRNAs were up‐regulated, and 2 were down‐regulated in the neoplasms studied compared to the control group, as illustrated in the heatmap (Figure S1).

Analysis of all neoplasms showed the highest up‐regulation levels for hsa‐miR‐373 (LogFC = 232.81), hsa‐miR‐675 (LogFC = 87.45), hsa‐miR‐508 (LogFC = 53.74), hsa‐miR‐1290 (LogFC = 47.31) and hsa‐miR‐483‐3p (LogFC = 37.89). In contrast, hsa‐miR‐105 (LogFC = −2.39) and hsa‐let‐7f‐1 (LogFC = −2.01) were the only down‐regulated miRNAs in the neoplasms studied compared to normal tissue (Table S2).

### 3.2 Differential miRNA Expression Between Malignant Neoplasms and Normal Glandular Tissue

When the malignant neoplasms ACC (*n* = 4) and MEC (*n* = 4) were compared with normal salivary gland tissues (*n* = 2), we identified 30 miRNAs with significant differential expression (*p* < 0.05), as shown in Table S3 and Figure S2.

All 30 miRNAs were up‐regulated in malignant tumours compared to normal tissue. Among the significantly up‐regulated miRNAs shared by both malignant tumour types were hsa‐miR‐373 (LogFC = 232.82; *p* = 0.045), hsa‐miR‐508 (LogFC = 75.93; *p* < 0.001), hsa‐miR‐1290 (LogFC = 66.96; *p* < 0.001) and hsa‐miR‐675 (LogFC = 65.49; *p* = 0.024) (Table S3).

Hierarchical cluster analysis showed that malignant neoplasms have a distinct miRNA profile when compared to normal tissue, with a clear separation between neoplasms and healthy tissue (Figure S2).

### 3.3 Differential miRNA Expression in Individual Salivary Gland Neoplasms Compared to Normal Glandular Tissue

**FIGURE 1:**
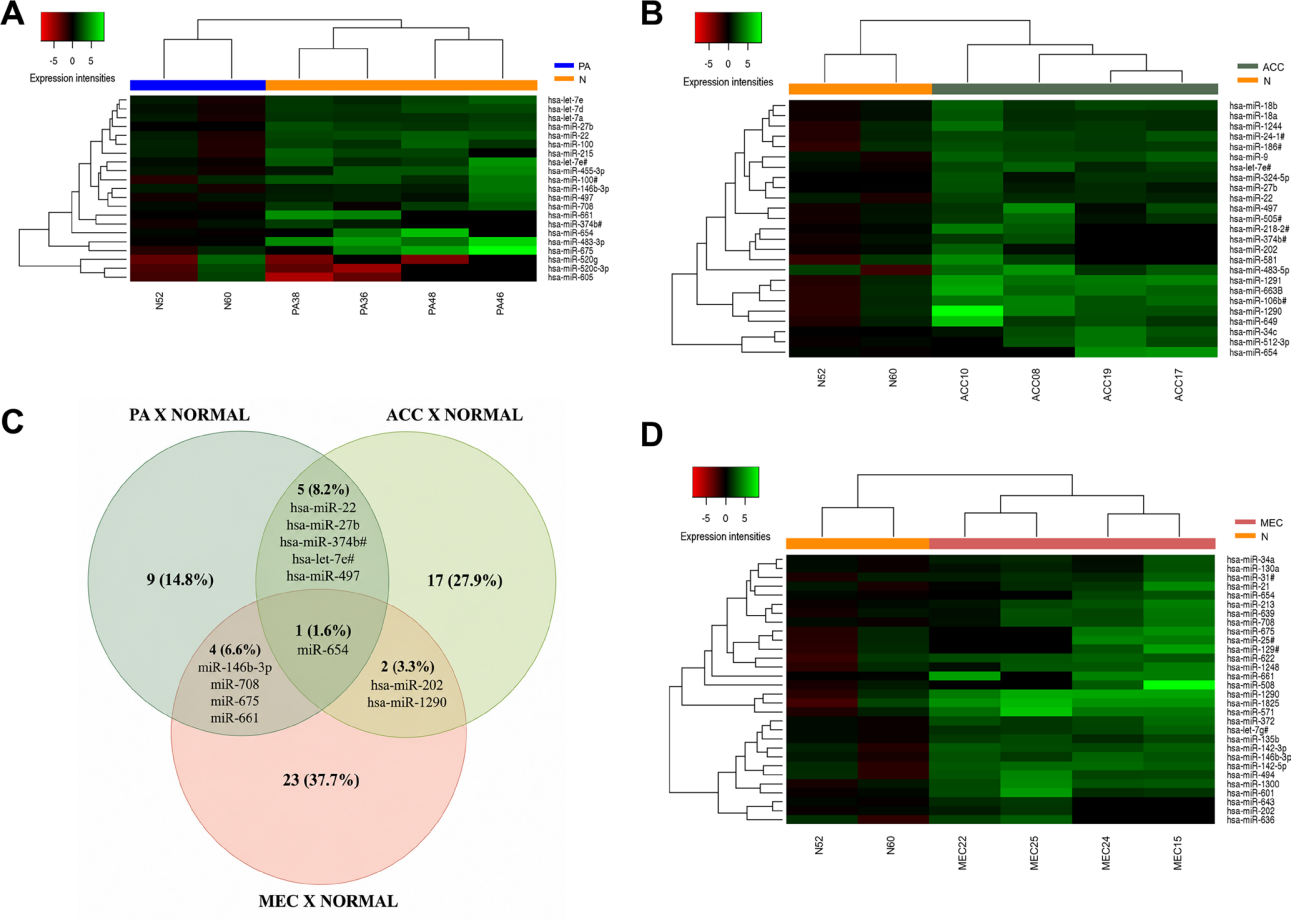
Hierarchical clustering of miRNAs differentially expressed in pleomorphic adenomas (PA) versus normal salivary glands (A), adenoid cystic carcinomas (ACC) versus normal salivary glands (B), and mucoepidermoid carcinomas (MEC) versus normal salivary glands (D). Venn diagram (C) illustrates differentially expressed miRNAs between salivary gland neoplasms and normal glandular tissue.

Comparison of individual salivary gland neoplasms with normal salivary gland tissues revealed differential expression (*p* < 0.05) of miRNAs in PA versus normal salivary glands (21 miRNAs), ACC versus normal salivary glands (25 miRNAs) and MEC versus normal salivary glands (30 miRNAs), as detailed in Table 1. In PA, 18 miRNAs were up‐regulated, and 3 were down‐regulated; whereas in ACC and MEC, all differentially expressed miRNAs were up‐regulated. These findings are illustrated in the heatmaps and Venn diagram of Figure 1. One miRNA, hsa‐miR‐654, was detected concomitantly across these three groups, with LogFC values of 45.45, 25.74 and 4.69, respectively.

**TABLE 1:** Differential miRNA expression in salivary gland neoplasms (PA, ACC and MEC) compared to normal glandular tissue.

PA versus N			ACC versus N			MEC versus N		
miRNA	Log fold change	*p*	miRNA	Log fold change	*p*	miRNA	Log fold change	*p*
hsa‐miR‐520 g	−4.59	0	hsa‐miR‐22	1.77	0	hsa‐miR‐202	1.54	0
hsa‐miR‐520c‐3p	−2.61	0	hsa‐miR‐27b	2.07	0	hsa‐miR‐130a	1.82	0
hsa‐miR‐605	−2.27	0	hsa‐miR‐324‐5p	2.63	0	hsa‐miR‐643	2.07	0
hsa‐miR‐374b#	1.82	0	hsa‐miR‐18a	2.82	0	hsa‐miR‐34a	2.07	0
hsa‐let‐7a	1.96	0	hsa‐miR‐186#	2.83	0	hsa‐miR‐31#	2.85	0
hsa‐miR‐708	2.84	0.047	hsa‐miR‐505#	3.55	0.047	hsa‐miR‐135b	3.10	0
hsa‐miR‐497	3.01	0.048	hsa‐miR‐374b#	3.74	0	hsa‐let‐7 g#	4.06	0
hsa‐let‐7d	3.13	0	hsa‐miR‐24‐1#	3.84	0	hsa‐miR‐654	4.69	0
hsa‐miR‐27b	3.38	0	hsa‐miR‐18b	4.06	0	hsa‐miR‐636	4.84	0
hsa‐let‐7e	3.40	0	hsa‐miR‐1244	4.07	0	hsa‐miR‐622	5.34	0
hsa‐miR‐100	3.69	0	hsa‐let‐7e#	4.10	0	hsa‐miR‐372	5.49	0
hsa‐miR‐146b‐3p	3.93	0	hsa‐miR‐202	4.12	0	hsa‐miR‐142‐3p	5.53	0
hsa‐miR‐215	4.64	0	hsa‐miR‐9	5.88	0	hsa‐miR‐146b‐3p	5.55	0
hsa‐miR‐22	5.20	0	hsa‐miR‐34c	6.06	0	hsa‐miR‐708	5.58	0
hsa‐miR‐100#	6.30	0	hsa‐miR‐497	7.31	0.047	hsa‐miR‐639	5.94	0.046
hsa‐miR‐455‐3p	8.10	0	hsa‐miR‐512‐3p	7.79	0	hsa‐miR‐21	6.66	0.048
hsa‐let‐7e#	9.22	0	hsa‐miR‐483‐5p	10.92	0.044	hsa‐miR‐213	6.89	0
hsa‐miR‐661	21.75	0	hsa‐miR‐218‐2#	10.97	0	hsa‐miR‐1248	7.02	0.046
hsa‐miR‐654	45.45	0	hsa‐miR‐106b#	11.58	0	hsa‐miR‐494	8.10	0
hsa‐miR‐483‐3p	45.88	0	hsa‐miR‐581	11.85	0	hsa‐miR‐142‐5p	8.22	0
hsa‐miR‐675	131.40	0	hsa‐miR‐663B	18.80	0	hsa‐miR‐1300	10.01	0
			hsa‐miR‐1291	20.27	0	hsa‐miR‐601	11.05	0
			hsa‐miR‐649	23.90	0	hsa‐miR‐25#	17.79	0
			hsa‐miR‐654	25.74	0	hsa‐miR‐675	20.25	0
			hsa‐miR‐1290	95.42	0	hsa‐miR‐129#	22.12	0
						hsa‐miR‐661	25.48	0
						hsa‐miR‐571	33.35	0
						hsa‐miR‐1825	37.21	0
						hsa‐miR‐1290	38.49	0
						hsa‐miR‐508	186.96	0

Abbreviations: ACC: adenoid cystic carcinoma; MEC: mucoepidermoid carcinoma; N: normal gland; PA: pleomorphic adenoma.

### 3.4 Differential miRNA Expression Between Benign and Malignant Neoplasms and Pairwise Tumour Comparisons

**FIGURE 2:**
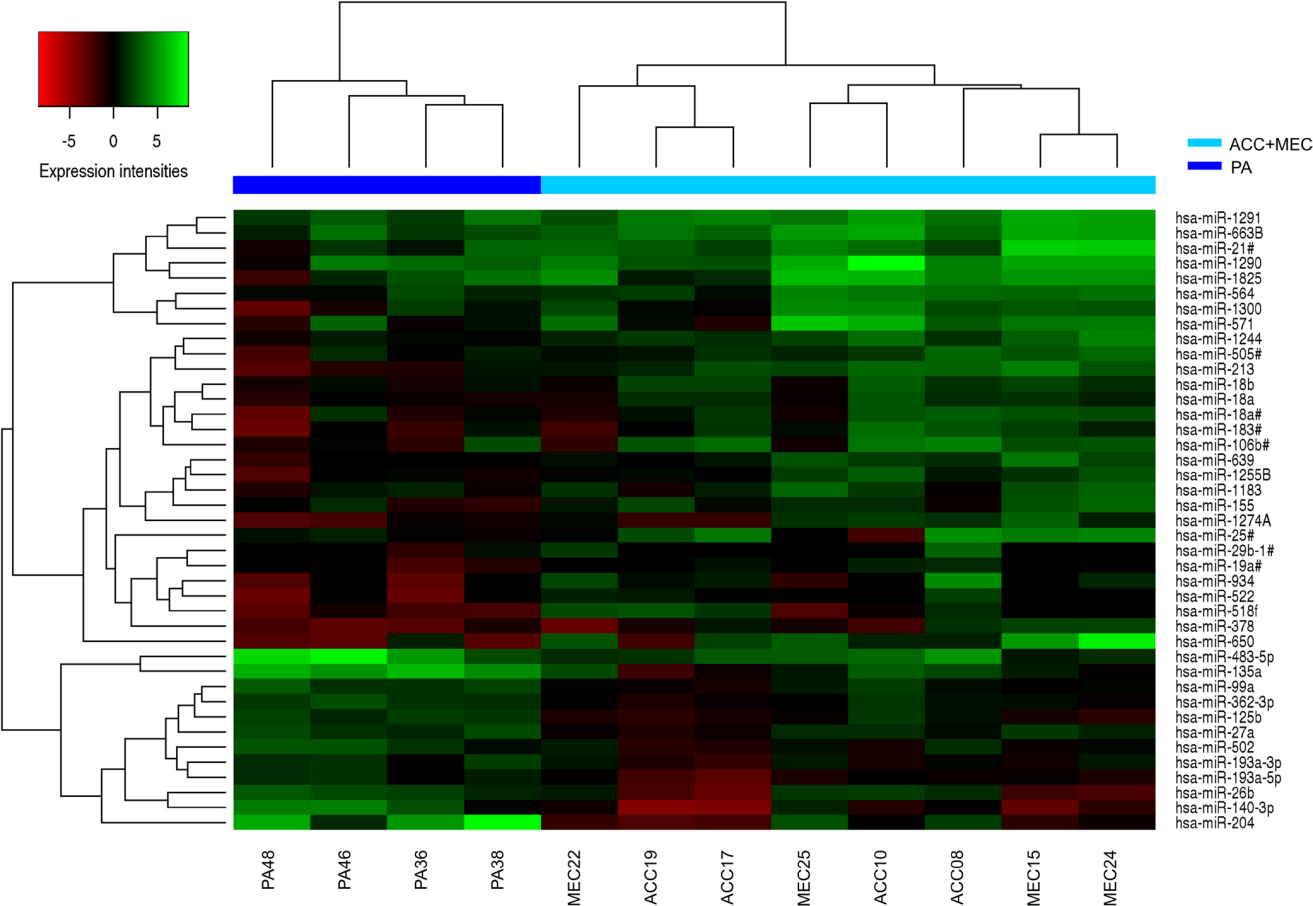
Hierarchical clustering of miRNAs differentially expressed in benign salivary gland neoplasms compared to malignant neoplasms. MicroRNAs with statistically significant differences were selected according to the variance between groups of interest. The distance between samples was determined based on the Euclidean distance and clustering was performed by complete linkage. The green colour represents overexpressed miRNAs and the red colour underexpressed miRNAs. The upper bar indicates cases of pleomorphic adenoma (PA) (black) and cases of mucoepidermoid carcinoma (MEC) and adenoid cystic carcinoma (ACC) (blue).

When benign (PA) and malignant (ACC and MEC) neoplasms were compared, we identified 42 miRNAs that were differentially expressed (*p* < 0.05), as shown in Table 2 and Figure 2. The miRNA hsa‐miR‐1290 showed the most pronounced negative Log fold change (−58.91) in benign neoplasms compared to malignant tumours. Significant variations were also observed for hsa‐miR‐21 (LogFC = −31.79) and hsa‐miR‐650 (LogFC = −38.48).

**TABLE 2:** Differential miRNA expression between malignant (ACC and MEC) and benign (PA) salivary gland neoplasms.

PA versus ACC, MEC		
miRNA	Log fold change	*p*
hsa‐miR‐1290	−58.912506	0.024
hsa‐miR‐650	−38.48249697	0.004
hsa‐miR‐21#	−31.7924978	0.038
hsa‐miR‐1825	−24.87	0.018
hsa‐miR‐571	−22.115	0.038
hsa‐miR‐663B	−20.1262503	0.01
hsa‐miR‐1291	−17.3487503	0.018
hsa‐miR‐25#	−12.8833344	0.046
hsa‐miR‐1300	−7.84875	0.018
hsa‐miR‐564	−7.7450012	0.037
hsa‐miR‐213	−6.86125046	0
hsa‐miR‐106b#	−6.3099999	0.034
hsa‐miR‐1244	−5.9537498	0.002
hsa‐miR‐29b‐1#	−5.6	0
hsa‐miR‐934	−5.52166703	0.031
hsa‐miR‐639	−4.43904805	0.002
hsa‐miR‐505#	−3.5875001	0.035
hsa‐miR‐1183	−3.21625	0.035
hsa‐miR‐183#	−3.20375	0.027
hsa‐miR‐18a#	−2.9324997	0.049
hsa‐miR‐518f	−2.85416682	0.029
hsa‐miR‐155	−2.7637501	0.036
hsa‐miR‐18b	−2.6887498	0.014
hsa‐miR‐1255B	−2.55125	0.008
hsa‐miR‐1274A	−2.4425	0.017
hsa‐miR‐522	−2.1225	0
hsa‐miR‐18a	−2.06	0.005
hsa‐miR‐19a#	−1.675	0
hsa‐miR‐378	−1.6412501	0.027
hsa‐miR‐193a‐5p	1.5275	0.004
hsa‐miR‐193a‐3p	1.6237502	0.041
hsa‐miR‐27a	2.2099998	0.05
hsa‐miR‐125b	2.47875	0.002
hsa‐miR‐362‐3p	2.5512501	0.019
hsa‐miR‐99a	3.14	0.034
hsa‐miR‐502	3.3125	0.043
hsa‐miR‐26b	3.4100003	0.045
hsa‐miR‐140‐3p	10.315	0.001
mmu‐miR‐140	16.66249994	0
hsa‐miR‐135a	42.2924999	0
hsa‐miR‐204	101.0275	0.001
hsa‐miR‐483‐5p	104.598749	0.043

Abbreviations: ACC: adenoid cystic carcinoma; MEC: mucoepidermoid carcinoma; PA: pleomorphic adenoma.

**FIGURE 3:**
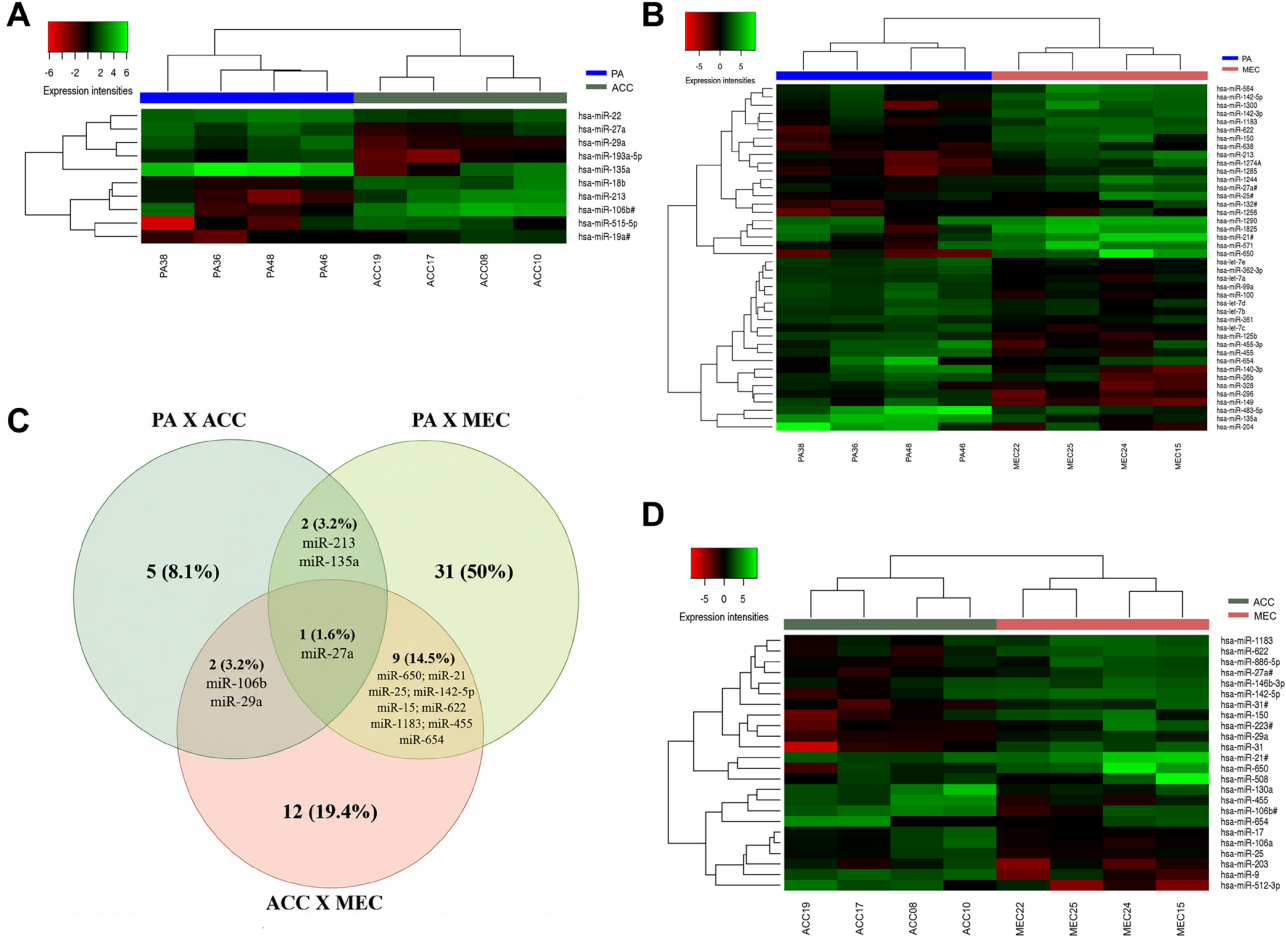
Hierarchical clustering of miRNAs differentially expressed in pleomorphic adenoma (PA) versus adenoid cystic carcinoma (ACC) (A), PA versus mucoepidermoid carcinoma (MEC) (B), and ACC versus MEC (D). Venn diagram (C) illustrating differentially expressed miRNAs between the salivary gland neoplasms.

Individual comparisons of salivary gland neoplasms (PA vs. ACC, PA vs. MEC and ACC vs. MEC) identified 78 miRNAs with statistically significant differences (*p* < 0.05), as shown in Table 3. The miRNAs differentially expressed (*p* < 0.05) between groups are illustrated in the heatmaps and Venn diagram of Figure 3. Hsa‐miR‐27a was the only miRNA that appeared concomitantly in the three groups of tumours.

**TABLE 3:** Differential miRNA expression in pairwise comparisons among salivary gland neoplasms.

PA versus ACC			PA versus MEC			ACC versus MEC		
miRNA	Log fold change	*p*	miRNA	Log fold change	*p*	miRNA	Log fold change	*p*
hsa‐miR‐106b#	−11.09	0.019	hsa‐miR‐650	−75.42	0.024	hsa‐miR‐508	−185.05	0
hsa‐miR‐213	−6.46	0.041	hsa‐miR‐21#	−60.69	0.029	hsa‐miR‐650	−73.88	0.033
hsa‐miR‐18b	−4.10	0.035	hsa‐miR‐1825	−33.96	0	hsa‐miR‐21#	−57.80	0.048
hsa‐miR‐515‐5p	−3.30	0.037	hsa‐miR‐571	−31.47	0	hsa‐miR‐142‐5p	−7.37	0
hsa‐miR‐19a#	−1.68	0.034	hsa‐miR‐1290	−30.45	0	hsa‐miR‐150	−6.91	0.03
hsa‐miR‐193a‐5p	1.61	0.036	hsa‐miR‐25#	−17.71	0	hsa‐miR‐223#	−6.40	0.007
hsa‐miR‐27a	2.68	0.035	hsa‐miR‐564	−9.81	0.033	hsa‐miR‐31	−6.18	0.017
hsa‐miR‐29a	3.17	0.037	hsa‐miR‐1300	−9.63	0.001	hsa‐miR‐622	−5.92	0
hsa‐miR‐22	3.44	0.013	hsa‐miR‐1244	−7.68	0.02	hsa‐miR‐1183	−5.32	0.022
hsa‐miR‐135a	41.76	0.032	hsa‐miR‐142‐5p	−7,54	0	hsa‐miR‐886‐5p	−4,29	0,034
			hsa‐miR‐213	−7,27	0	hsa‐miR‐146b‐3p	−4,12	0,026
			hsa‐miR‐150	−7,10	0	hsa‐miR‐31#	−3.44	0.003
			hsa‐miR‐622	−6.42	0	hsa‐miR‐27a#	−3.18	0.018
			hsa‐miR‐1183	−5.88	0	hsa‐miR‐29a	−1.78	0.013
			hsa‐miR‐142‐3p	−5.24	0.016	rno‐miR‐29c#	−1.64	0
			hsa‐miR‐1274A	−3.50	0	hsa‐miR‐203	1.73	0.039
			hsa‐miR‐638	−3.31	0	hsa‐miR‐25	1.74	0.037
			hsa‐miR‐27a#	−2.78	0.039	hsa‐miR‐17	2.77	0
			hsa‐miR‐132#	−2.36	0	hsa‐miR‐106a	3.33	0
			hsa‐miR‐1256	−1.74	0	hsa‐miR‐9	6.06	0
			hsa‐miR‐1285	−1.66	0.043	hsa‐miR‐512‐3p	8.11	0
			hsa‐miR‐328	1.58	0.026	hsa‐miR‐106b#	9.57	0.028
			hsa‐miR‐296	1.65	0.002	hsa‐miR‐455	12.92	0
			hsa‐miR‐361	1.72	0.047	hsa‐miR‐654	21.05	0
			hsa‐let‐7a	1.90	0	hsa‐miR‐130a	23.85	0.047
			hsa‐let‐7d	2.03	0.019			
			hsa‐let‐7c	2.24	0.004			
			hsa‐miR‐362‐3p	2.83	0.017			
			hsa‐miR‐125b	2.93	0			
			hsa‐let‐7b	3.00	0.027			
			hsa‐let‐7e	3.26	0			
			hsa‐miR‐99a	3.36	0.016			
			hsa‐miR‐26b	3.58	0.028			
			hsa‐miR‐149	3.80	0.011			
			hsa‐miR‐100	4.08	0			
			hsa‐miR‐455	5.62	0.007			
			hsa‐miR‐455‐3p	7.18	0.035			
			hsa‐miR‐140‐3p	10.08	0.028			
			mmu‐miR‐140	16.37	0			
			hsa‐miR‐654	40.76	0			
			hsa‐miR‐135a	42.83	0.007			
			hsa‐miR‐204	100.70	0.026			
			hsa‐miR‐483‐5p	109.28	0.035			

Application of ANOVA revealed the following miRNAs to be significantly differentially expressed (*p* < 0.05) in each neoplasm compared to normal salivary gland tissue: hsa‐miR‐106b, hsa‐miR‐142‐5p, hsa‐miR‐622, hsa‐miR‐642, hsa‐miR‐574‐3p and hsa‐miR‐486 (Figure S3).

## 4 Discussion

This study revealed distinct miRNA expression profiles in MECs and ACCs compared to PAs, with the detection of 42 that distinguished these groups of neoplasms. Likewise, all benign and malignant neoplasms studied exhibited significant differences compared to normal salivary gland tissue, highlighting the potential of miRNAs in the development of salivary gland neoplasms and tumourigenesis. Therefore, our results provide a comprehensive molecular signature of these tumours.

The present study evaluated 754 miRNAs in three types of salivary gland neoplasms; of these, 29 were significantly differentially expressed when benign and malignant neoplasms were grouped and compared with normal salivary gland tissue samples. Among the down‐regulated miRNAs, hsa‐miR‐105 and hsa‐let‐7f‐1 are highlighted, while hsa‐miR‐373, hsa‐miR‐675, hsa‐miR‐508, hsa‐miR‐1290 and hsa‐miR‐483‐3p were up‐regulated when compared to normal salivary gland tissue. Interestingly, the same miRNAs were also up‐regulated when ACC and MEC were analysed together and compared to normal salivary gland tissue. In contrast, other authors have reported up‐regulation of miR‐1271‐5p in ACC samples [9], while miR375 and miR99b were down‐regulated, and miR140 was up‐regulated in PA [10]. Another study identified miR133b and miR99b to be up‐regulated in different salivary gland tumours, including ACC and MEC, while let7a and miR140 were down‐regulated [11]. However, up‐regulation of miR140 in ACC samples has also been reported [6]. In our study, miR‐21 was differentially expressed between groups when MEC and normal salivary gland were compared, which is in line with the findings of Zanon et al. (2023), who also reported miR‐21 overexpression in ACC. These findings show that miRNAs can be used to distinguish salivary gland neoplasms from the tissue that gave rise to them and how complex the molecular profile of these neoplasms is in different studies.

It is important to highlight the role of miR‐21 in the progression of ACC. Studies have demonstrated the overexpression of miR‐21 in metastatic ACC, which correlates with high levels of cell migration and invasion [12]. This miRNA was also found to down‐regulate tumour suppressor genes such as *PTEN*, *PDCD4* and *BCL‐2*, promoting cell survival and contributing to treatment resistance. In ACC cells with high metastatic potential, the reduction in miR‐21 increased the expression of these suppressor genes and decreased anti‐apoptotic protein BCL‐2 [13]. Furthermore, miR‐21 is involved in epithelial‐mesenchymal transition (EMT), an essential process for invasion and metastasis, and its inhibition has been shown to restore epithelial characteristics in metastatic ACC cells [14]. Taken together, these findings indicate miR‐21 as a promising biomarker for the diagnosis of salivary gland neoplasms, especially ACC, and as a potential therapeutic target, particularly for more aggressive and treatment‐resistant neoplasms.

Although hsa‐miR‐373 was up‐regulated, this result was observed in only one sample of ACC. This did not occur with MEC. This miRNA is related to human embryonic stem cells [15] and participates in essential cellular processes such as proliferation, apoptosis, senescence, migration and invasion in several types of cancer [16]. Other authors found hsa‐miR‐373 to be up‐regulated in squamous cell carcinoma, MEC, PA and Warthin's tumour compared to normal salivary gland, with the up‐regulation of this miRNA being associated with cases without perineural invasion and with metastasis; however, ACC was not studied [17]. In contrast, all ACC cases in our study exhibited perineural invasion. It is possible that the ACC case of the present study, in which hsa‐miR‐373 was up‐regulated, was highly aggressive, as indicated by the presence of solid and cribriform areas. Cancers in which this miRNA is up‐regulated are characterised by greater resistance to cisplatin chemotherapy [18].

Hsa‐miR‐675 is present exclusively in post‐functional placenta [19], and its dysregulation is involved in multiple steps of carcinogenesis in different types of cancers, including gastrointestinal carcinomas [20]. To our knowledge, the presence of this miRNA in salivary gland neoplasms has not been reported in previous studies, although it is commonly found in gastrointestinal carcinomas [20, 21]. Hsa‐miR‐675 was unable to distinguish PA from the malignant neoplasms studied since it was up‐regulated in all cases. This miRNA was also found to be up‐regulated in gastric cancer cell lines compared to normal tissues, in which its expression was associated with metastasis and induction of EMT through an increase in the expression of N‐cadherin, vimentin and MMP‐9 and a decrease in the expression of E‐cadherin [22]. This alteration is consistent with the role of hsa‐miR‐675 in the promotion of cell migration and invasion. In a previous study, we detected positive correlations between N‐cadherin/SNAIL in ACC, SNAIL/TWIST in MEC and SLUG/TWIST in PA [23].

The hsa‐miR‐508 regulates multiple genes related to EMT, influencing different cell types [24]. Notably, the up‐regulation of hsa‐miR‐508 in one PA sample, combined with its established oncogenic role in other types of cancer, suggests its potential role as an oncogene in certain salivary gland neoplasms. Its oncogenic role is supported by studies investigating other types of cancer, such as ovarian cancer, in which high hsa‐miR‐508 expression was associated with an un‐favourable prognosis [25].

Similarly, hsa‐miR‐1290 was also up‐regulated in the samples of malignant neoplasms when compared to both normal salivary gland tissue and PA. We highlight one case of solid ACC that exhibited higher expression levels of hsa‐miR‐1290 compared to the other histological subtypes of this tumour. In addition, this miRNA was highly expressed and distinctly regulated in MEC when compared to PA. Studies indicated hsa‐miR‐1290 to be an important biomarker of pancreatic adenocarcinoma, colorectal cancer and cervical cancer [26, 27].

The hsa‐miR‐483‐3p participates in key cellular processes such as cell growth, migration and invasion, which are essential for tumour progression. Here, hsa‐miR‐483‐3p was up‐regulated in all neoplasms analysed, including PA, when compared to normal salivary gland tissue, suggesting a potential role of this miRNA in salivary gland tumourigenesis. These results are interesting and appear complex, as overexpression of hsa‐miR‐483‐3p had the opposite effect in seminoma cell lines, inhibiting cell growth, migration and invasion [28]. In contrast, hsa‐miR‐483‐3p expression correlated positively with hepatocellular carcinomas and negatively with non‐neoplastic liver tissues [29].

We highlight hsa‐miR‐105 and hsa‐let‐7f‐1 as the two least expressed miRNAs in the neoplasms studied here when compared to normal salivary gland tissues. These miRNAs probably act as tumour suppressors, similar to reports for hepatocellular carcinoma. However, although hsa‐miR‐105 is associated with tumour suppression, previous studies indicate an oncogenic role in colorectal cancer [25]. Furthermore, hsa‐miR‐105 was found to be up‐regulated in oral squamous cell carcinoma when compared to the control group, indicating that the higher its expression, the more advanced the tumour (T3 and T4) [25]. In contrast, hsa‐let‐7f‐1, which belongs to the let‐7 family of miRNAs, was up‐regulated in colorectal cancer [30].

It is important to stress that this study shows some limitations. The results were based on a small sample of salivary gland tumours, although adenoid cystic carcinoma and MEC are rare tumours. Also, we were unable to analyse the clinical features and their histopathological patterns, as these tumours showed significant variation in these aspects. Therefore, the present investigation should be interpreted as a pilot study providing preliminary evidence of miRNA dysregulation in salivary gland neoplasms. Further studies, including larger, independent cohorts and functional validation experiments, are warranted to confirm the biological significance and potential clinical applicability of the identified miRNA signatures.

## 5 Conclusion

In the present study, we describe the global molecular signature of miRNAs in salivary gland neoplasms. Differential expression of miRNAs was observed in 12 cases of salivary gland neoplasms, including benign and malignant tumours, when compared to normal salivary gland tissue. These findings provide preliminary evidence of miRNA dysregulation in salivary gland neoplasms and suggest a potential association between specific miRNA signatures and molecular pathways implicated in tumour progression.

## Author Contributions

**Ana Carolina Velasco Pondé de Sena:** writing – original draft, visualisation, investigation, data curation. **Poliana Ramos Braga:** writing – original draft, visualisation, investigation, data curation. **Flávia Caló de Aquino Xavier:** writing – review and editing, investigation, data curation. **Dandara Andrade de Santana:** writing – review and editing, investigation, data curation. **Fernando Augusto Soares:** writing – review and editing. **Iguaracyra Barreto de Araújo:** writing – review and editing. **Patricia Ramos Cury:** writing – review and editing. **Daniel Araki Ribeiro:** writing – review and editing. **Fabio Albuquerque Marchi:** writing – review and editing, investigation. **Valéria Souza Freitas:** writing – review and editing, investigation. **Cláudia Malheiros Coutinho‐Camillo:** writing – review and editing, investigation. **Jean Nunes dos Santos:** formal analysis, investigation, project administration, supervision, writing – original draft, writing – review and editing.

## Funding

This work was supported by Conselho Nacional de Desenvolvimento Científico e Tecnológico. Coordenação de Aperfeiçoamento de Pessoal de Nível Superior.

## Ethics Statement

This study was approved by the Ethics Committee of the School of Dentistry, Federal University of Bahia (FOUFBA; Opinion number 487.2995.1). All procedures followed the ethics standards of the Helsinki Declaration of 1975, as revised in 2008. This article does not contain any studies with human or animal subjects performed by any of the authors.

## Conflicts of Interest

The authors declare no conflicts of interest.

## Supporting information


**Figure S1:** Hierarchical clustering of miRNAs differentially expressed in salivary gland neoplasms compared to normal glandular tissue. MicroRNAs with statistically significant differences were selected according to the variance between groups of interest. The distance between samples was determined based on the Euclidean distance, and clustering was performed by complete linkage. The green colour represents overexpressed miRNAs, and the red colour represents underexpressed miRNAs. The upper bar indicates normal samples (black) and samples of salivary gland neoplasms (pleomorphic adenoma [PA], adenoid cystic carcinoma [ACC] and mucoepidermoid carcinoma [MEC]) (blue).

**Figure S2:** Hierarchical clustering of miRNAs differentially expressed in malignant salivary gland neoplasms compared to normal glandular tissue. MicroRNAs with statistically significant differences were selected according to the variance between groups of interest. The distance between samples was determined based on the Euclidean distance and clustering was performed by complete linkage. The green colour represents overexpressed miRNAs and the red colour underexpressed miRNAs. The upper bar indicates normal samples (black) and samples of malignant salivary gland neoplasms (adenoid cystic carcinoma [ACC] and mucoepidermoid carcinoma [MEC]) (blue).

**Figure S3:** Hierarchical clustering of miRNAs differentially expressed in adenoid cystic carcinomas (ACC) mucoepidermoid carcinomas (MEC) and pleomorphic adenoma (PA) compared to normal salivary gland tissue. MicroRNAs with statistically significant differences were selected according to the variance between groups of interest. The distance between samples was determined based on the Euclidean distance and clustering was performed by complete linkage. The green colour represents overexpressed miRNAs and the red colour underexpressed miRNAs. The upper bar indicates cases of ACC (blue) and MEC (black).

**Table S1:** Clinical data of the tumours and control cases.

**Table S2:** Differential miRNA expression between salivary gland neoplasms (PA, ACC and MEC) and normal glandular tissue.

**Table S3:** Differential miRNA expression between malignant salivary gland neoplasms (ACC and MEC) and normal glandular tissue.

## Data Availability

The data that support the findings of this study are available from the corresponding author upon reasonable request.
