# Supplementary material for: Heterogeneity of miRNAs in Salivary Gland Neoplasms: An Exploratory Pilot Study
Source: J Oral Pathol Med. 2026 May 25;55(8):906–16. doi: 10.1111/jop.70150 (PMC13533855; doi:10.1111/jop.70150)
Supplement: Supplementary file 1 [file JOP-55-906-s003.docx]

| **Supplementary Table 3.** Differential miRNA expression between malignant salivary gland neoplasms (ACC and MEC) and normal glandular tissue | | |
| --- | --- | --- |
| **ACC, MEC vs Normal** | | |
| **miRNA** | **Log fold change** | **p-value** |
| hsa-miR-515-5p | 1.925 | 0.045 |
| hsa-miR-202 | 2.8275 | 0.027 |
| hsa-miR-374b# | 3.28 | 0.04 |
| hsa-let-7g# | 3.64375 | 0.041 |
| hsa-miR-518c | 3.715 | 0.042 |
| hsa-miR-639 | 4.085715 | 0.022 |
| hsa-miR-22 | 4.18375 | 0.034 |
| hsa-miR-186# | 4.59875 | 0.041 |
| hsa-miR-29b-1# | 5.295 | 0 |
| hsa-let-7i# | 5.375 | 0.042 |
| hsa-miR-1244 | 5.79375 | 0.045 |
| hsa-miR-213 | 6.481251 | 0 |
| hsa-miR-494 | 7.0525 | 0.017 |
| hsa-miR-218-2# | 7.068333 | 0.05 |
| hsa-miR-564 | 9.215001 | 0.027 |
| hsa-miR-129# | 12.622 | 0.035 |
| hsa-miR-581 | 12.93 | 0.027 |
| hsa-miR-25# | 12.96 | 0.049 |
| hsa-miR-130a | 13.7375 | 0 |
| hsa-miR-654 | 15.2125 | 0.031 |
| hsa-miR-649 | 19.00875 | 0 |
| hsa-let-7c# | 19.91 | 0.031 |
| hsa-miR-1291 | 23.46625 | 0.028 |
| hsa-miR-663B | 24.81625 | 0.027 |
| hsa-miR-21# | 33.5675 | 0.043 |
| hsa-miR-483-3p | 33.90875 | 0.024 |
| hsa-miR-675 | 65.48667 | 0.024 |
| hsa-miR-1290 | 66.95501 | 0 |
| hsa-miR-508 | 75.932 | 0 |
| hsa-miR-373 | 232.8167 | 0.045 |
| ACC: adenoid cystic carcinoma; MEC: mucoepidermoid carcinoma. | | |
