# Supplementary material for: Heterogeneity of miRNAs in Salivary Gland Neoplasms: An Exploratory Pilot Study
Source: J Oral Pathol Med. 2026 May 25;55(8):906–16. doi: 10.1111/jop.70150 (PMC13533855; doi:10.1111/jop.70150)
Supplement: Supplementary file 2 [file JOP-55-906-s005.docx]

| **Supplementary Table 2.** Differential miRNA expression between salivary gland neoplasms (PA, ACC and MEC) and normal glandular tissue | | |
| --- | --- | --- |
| **ACC, MEC, PA vs Normal** | | |
| **miRNA** | **Log fold change** | **p-value** |
| hsa-miR-105 | -2.39 | 0.015 |
| hsa-let-7f-1# | -2.01833343 | 0.027 |
| hsa-miR-548a | 2.045 | 0 |
| hsa-miR-27b | 2.0491667 | 0.037 |
| hsa-miR-29b-1# | 2.4949999 | 0.043 |
| hsa-miR-374b# | 2.7916667 | 0.02 |
| hsa-miR-744 | 2.8675 | 0.038 |
| hsa-miR-490 | 3.2149999 | 0.014 |
| hsa-miR-526b | 3.5274997 | 0.049 |
| hsa-miR-146b-3p | 3.6349996 | 0.021 |
| hsa-miR-202 | 3.6362506 | 0.033 |
| hsa-miR-518c | 3.7150002 | 0.038 |
| hsa-miR-218-2# | 4.497 | 0.05 |
| hsa-miR-22 | 4.523333 | 0.014 |
| hsa-let-7e# | 6.561667 | 0.01 |
| hsa-miR-564 | 6.633333 | 0.028 |
| hsa-miR-581 | 7.465999 | 0.036 |
| hsa-miR-130a | 10.469167 | 0.004 |
| hsa-miR-494 | 11.050001 | 0.035 |
| hsa-miR-649 | 13.555 | 0.009 |
| hsa-miR-1291 | 17.683333 | 0.035 |
| hsa-let-7c# | 17.7007139 | 0.033 |
| hsa-miR-654 | 25.29167 | 0 |
| hsa-miR-661 | 26.781665 | 0.039 |
| hsa-miR-483-3p | 37.899167 | 0.008 |
| hsa-miR-1290 | 47.3175 | 0.009 |
| hsa-miR-508 | 53.7475 | 0.013 |
| hsa-miR-675 | 87.456664 | 0 |
| hsa-miR-373 | 232.81668 | 0.03 |
| ACC: adenoid cystic carcinoma; MEC: mucoepidermoid carcinoma; PA: pleomorphic adenoma. | | |
