# Supplementary material for: Heterogeneity of miRNAs in Salivary Gland Neoplasms: An Exploratory Pilot Study
Source: J Oral Pathol Med. 2026 May 25;55(8):906–16. doi: 10.1111/jop.70150 (PMC13533855; doi:10.1111/jop.70150)
Supplement: Supplementary file 3 [file JOP-55-906-s006.docx]

| **Supplementary Table 1**: Clinical data of the tumours and control cases. | | | | | | |
| --- | --- | --- | --- | --- | --- | --- |
| **Tumour** | **Case** | **Pattern/Histological grade** | **Gender** | **Age  (years)** | **Anatomic location** | **Size (cm)** |
| PA | 1 | Classic | F | 67 | Parotid | 5.50×3.50×2.00 |
| PA | 2 | Classic | F | 59 | Soft palate | 4.00×2.80×2.20 |
| PA | 3 | Classic | F | 55 | Submandibular | 5.00×4.00×3.00 |
| PA | 4 | Classic | M | 59 | Hard palate | 2.60×2.20×1.90 |
| ACC | 5 | Cribriform | M | 42 | Oral mucosa | 8.00×6.00×2.50 |
| ACC | 6 | Cribriform with solid areas | F | 53 | Submandibular | 2.40×1.60×1.40 |
| ACC | 7 | Cribriform | M | 59 | Upper lip | 3.90×2.70×1.40 |
| ACC | 8 | Solid | M | 78 | Parotid | 4.50×3.00×2.70 |
| MEC | 9 | Low grade | F | 51 | Parotid | 6.50×4.50×3.00 |
| MEC | 10 | Intermediate grade | M | 22 | Parotid | 6.50×5.50×5.00 |
| MEC | 11 | Low grade | M | 29 | Parotid | 3.50×3.20×2.80 |
| MEC | 12 | Intermediate grade | F | 44 | Parotid | 6.00×5.00×1.70 |
| N | 13 | - | F | 31 | Buccal mucosa | 2.50×0.30×0.10 |
| N | 14 | - | F | 34 | Buccal mucosa | 0.90×0.40×0.10 |
| PA: pleomorphic adenoma, ACC: adenoid cystic carcinoma, MEC: mucoepidermoid carcinoma, N: normal gland. F: female, M: male. | | | | | | |
